# Dealing with Psychotic Symptoms at Digital Distance

**DOI:** 10.1192/j.eurpsy.2022.182

**Published:** 2022-09-01

**Authors:** W. Gaebel

**Affiliations:** LVR-Klinikum , Psychiatry, Düsseldorf, Germany

**Keywords:** eMental health interventions, psychotic disorders

## Abstract

The EPA Guidance on the Quality of eMental health interventions in the treatment of psychotic disorders (1), based on systematic literature review, found strong evidence that web- and mobile based interventions for people with schizophrenia and/or other psychotic disorders are feasible and acceptable both for patients and caregivers. There was moderate evidence that eMental health interventions may improve specific elements of mental healthcare processes, such as shared-decision-making, symptom monitoring, disease management, information provision, empowerment, and there was preliminary evidence that they may also improve outcomes by fostering symptom reduction and treatment adherence. E-mental health interventions hold promise to shape the future of mental healthcare delivery through increasing service accessibility, reducing stigma and self-stigma, and providing timely and flexible support to individuals with psychotic disorders and their caregivers. Nevertheless, it is important to also consider other aspects such as the lack of ethical guidelines and quality assurance mechanisms, and the need to analyse the legal frameworks about eMental health in different nations when developing and implementing eMental health interventions. We did not identify ethical guidelines or quality assurance systems specifically developed for eMental health interventions targeting people with psychotic disorders. E-mental health interventions are efficacious to increase mental health literacy. We also found preliminary evidence that eMental health interventions are efficacious to treat psychotic disorders. Recent overviews (2) are coming to similar conclusions. Future research needs to provide better controlled, sufficiently powered studies to provide definite answers to open questions. Gaebel et al., 2016, DOI 10.1007/s00406-016-0677-6 Donahue, Rodriguez, Shore, 2021, doi.org/10.1007/s11920-021-01242-y

**Disclosure:**

No significant relationships.

